# N7-Methylguanosine Modification in Colorectal Cancer: Molecular Insights and Clinical Implications

**DOI:** 10.3390/ijms27125228

**Published:** 2026-06-09

**Authors:** Qin Zhang, Chunchun Li, Yonglan Zhu, Meirong Yu, Yanshan Liu, Yuqiong Xie, Jiang Cao

**Affiliations:** Center for Basic Research, The Second Affiliated Hospital, School of Medicine, Zhejiang University, Hangzhou 310009, China; zhangxqin@zju.edu.cn (Q.Z.); lichunchun@zju.edu.cn (C.L.); zhuyonglan@zju.edu.cn (Y.Z.); 2519125@zju.edu.cn (M.Y.); 21718007@zju.edu.cn (Y.L.)

**Keywords:** N7-methylguanosine, epigenetic modification, METTL1, eIF4E, colorectal cancer

## Abstract

Colorectal cancer (CRC) remains a leading cause of cancer-related mortality worldwide, characterized by a multi-step carcinogenesis process involving genetic mutations and epigenetic alterations. Despite advances in screening and therapy, challenges such as treatment resistance, recurrence, and metastasis persist. Emerging evidence highlights the critical role of epigenetic modifications, particularly N7-methylguanosine (m7G), in post-transcriptional regulation. This ubiquitous RNA modification participates extensively in tumor biological behaviors by regulating RNA stability, processing, and translation. Studies have shown that dysregulation of m7G modification is closely associated with adverse clinical outcomes in CRC. This review systematically summarizes the biological functions of m7G modification and its key regulatory proteins (such as METTL1/WDR4, eIF4E, etc.), with a focus on their roles in the pathogenesis, progression, prognosis, and diagnosis of, as well as therapy for, colorectal cancer. m7G modification and related molecules hold potential as novel biomarkers and therapeutic targets, thereby providing new strategies for the precision diagnosis and treatment of colorectal cancer.

## 1. Introduction

Colorectal cancer (CRC) ranks as the third most commonly diagnosed malignancy and a leading cause of cancer-related mortality worldwide [1]. While screening colonoscopies have contributed to a declining incidence in older populations, a concerning global increase in early-onset CRC among individuals under 50 presents a pressing public health challenge [2]. The primary management of CRC involves surgery-centered multimodal therapy, supplemented by chemotherapy, radiotherapy, targeted therapy, and immunotherapy. However, significant therapeutic hurdles persist. Surgery offers limited benefit for metastatic or unresectable tumors; chemotherapy is hampered by severe side effects, drug resistance, and recurrence; and radiotherapy may elevate the risk of secondary malignancies [3]. Although targeted therapies offer specificity for advanced cases, their efficacy is often curtailed by the development of resistance through on-target mutations or bypass pathway activation [4]. Similarly, immunotherapy demonstrates minimal efficacy as a single agent in the majority of CRC cases, which are microsatellite stable (MSS) or mismatch repair proficient (pMMR) [5]. The pathogenesis of CRC is highly complex, involving multifactorial elements that remain incompletely understood. Therefore, a deeper investigation into the molecular mechanisms driving CRC initiation and progression, alongside the identification of novel diagnostic biomarkers and therapeutic targets, is crucial for advancing targeted therapies and improving treatment outcomes.

CRC research has entered the era of precision medicine, defined by molecular subtyping. Epigenetic modifications, which regulate gene expression without altering the DNA sequence, provide critical insights into tumor heterogeneity, malignant progression, and therapeutic resistance [6]. These modifications include DNA methylation, histone modifications, and RNA methylation, with growing evidence highlighting the predominant role of RNA methylation in cancer biology [7,8]. High-throughput sequencing has revealed over 170 RNA modifications. Among these, N7-methylguanosine (m7G) has emerged as a key post-transcriptional regulator. Unlike neutral N6-methyladenosine (m6A) or 5-methylcytidine (m5C), m7G carries a permanent positive charge and uniquely exists both as the 5′ cap and as an internal modification, installed by distinct writers. Furthermore, it lacks a validated eraser, is far less abundant, and regulates multiple layers of RNA metabolism, from translation to tRNA stability [9].

The m7G modification involves methylation at the N7 position of guanosine, catalyzed by specific methyltransferases [10]. As one of the most abundant and evolutionarily conserved RNA modifications, m7G is present across various RNA species, where it fulfills diverse biological functions. Initially identified in the 5′ cap of messenger RNAs (mRNAs), where it regulates nuclear export, stability, and translation initiation [11], m7G has also been found internally within mRNAs, dynamically modulating translation efficiency under stress conditions [12]. In transfer RNAs (tRNAs), m7G is crucial for structural stability [13], and in ribosomal RNAs (rRNAs), it facilitates ribosome maturation and assembly [14]. Dysregulation of m7G modification is implicated in the pathogenesis of multiple diseases, including neurological disorders [15], cardiovascular conditions [16], skeletal muscle system diseases [17], and various cancers [10]. Accumulating evidence specifically links aberrant m7G modification to poor clinical prognosis in CRC patients [18]. This review summarizes the biological functions of m7G and its regulatory proteins, with a focused discussion on their key roles in the pathogenesis, progression, prognosis, and treatment of colorectal cancer. We aim to provide new perspectives for advancing CRC diagnosis and targeted therapy.

## 2. Regulatory Proteins and Biological Functions of m7G Modification

In RNA methylation, three key classes of regulatory factors typically orchestrate dynamic and reversible modifications: writers (methyltransferases) that catalyze modifications, erasers (demethylases) that remove them, and readers (binding proteins) that recognize and bind to modified sites [19]. These proteins collectively fine-tune gene expression by modulating the stability, accessibility, availability and function of most kinds of RNAs and, by regulating specific modifications across diverse RNA substrates, are integral to normal cellular function and metabolism.

### 2.1. Writers

**Figure 1:**
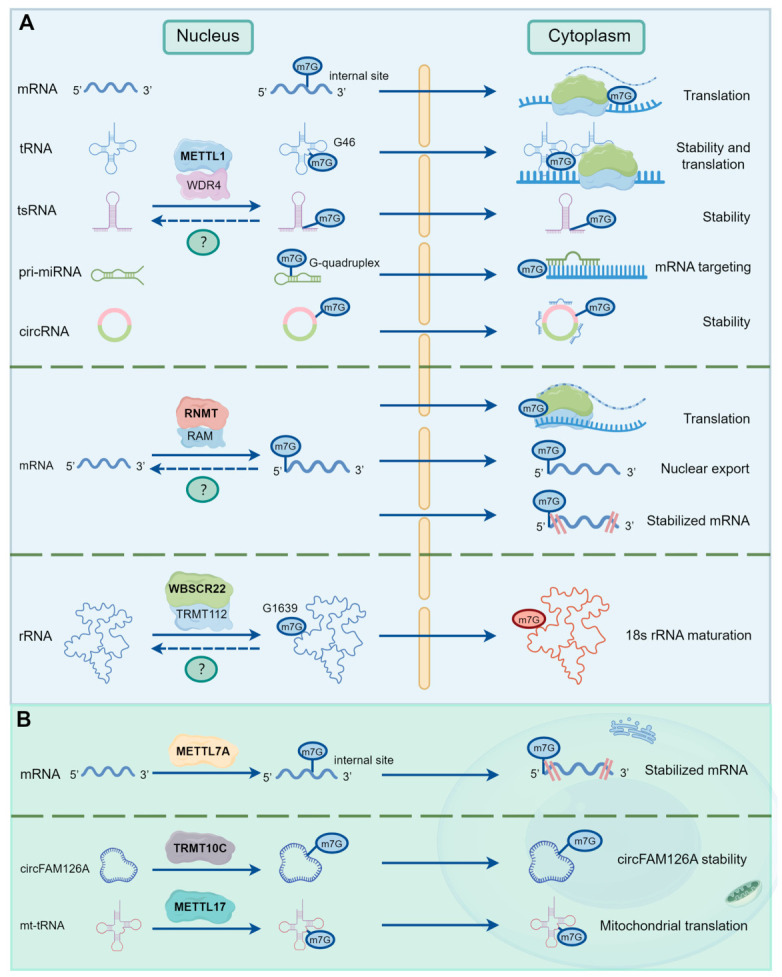
RNA m7G modifications mediated by methyltransferases (writers) and their biological functions in various RNA types. (Created with Figdraw; Export ID: RWUAA51a55, www.figdraw.com (accessed on 6 May 2026).) (**A**) The left panel (nucleus) depicts the key writers, including METTL1/WDR4, RNMT/RAM and WBSCR22/TRMT112, that catalyze m7G modification at distinct positions on target RNA molecules, while the reversibility of m7G modification (shown in dashed lines) remains unknown. The right panel (cytoplasm) illustrates the significant cytological functions of m7G, including RNA stability, processing, and translation. (**B**) The left panel depicts the non-classical regulators of m7G modification, including METTL7A, TRMT10C and METTL17. The right panel shows the biological effects on subcellular localization.

Several evolutionarily conserved methyltransferase complexes catalyze m7G modification in mammals, including METTL1/WDR4, RNMT/RAM, and WBSCR22/TRMT112 (Figure 1A). Each complex installs m7G at distinct positions on target RNAs, thereby conferring unique functional outcomes.

#### 2.1.1. METTL1/WDR4

Methyltransferase-like 1 (METTL1) is the most extensively characterized m7G methyltransferase. It functions as a heterodimeric complex with WD repeat domain 4 (WDR4), where METTL1 serves as the catalytic core. This complex transfers a methyl group from S-adenosyl methionine (SAM) to guanosine, producing S-adenosylhomocysteine (SAH) and m7G-modified RNA. Its substrates include internal sites within mRNA, tRNA at position G46, and G-quadruplex structures in microRNA (miRNA).

Function in mRNA: METTL1/WDR4 catalyzes low-abundance internal m7G modifications (∼0.02–0.05%) within the 5′ untranslated region (UTR), coding sequence (CDS), and 3′ UTR of mRNAs, thereby influencing translation [12]. Under heat shock and oxidative stress, m7G enrichment in the CDS and 3′ UTR dynamically increases [20]. These modifications promote recruitment of target mRNAs to stress granules SGs; METTL1 knockdown reduces m7G abundance within SGs, impairing mRNA stability and translational regulation [21]. In cancer cells, 3′ UTR m7G promotes degradation of transcripts involved in cell cycle, mRNA regulation, and chromatin remodeling [22]. The functional outcomes are cell-type-specific: in neural stem cells, METTL1/WDR4-mediated m7G enhances the stability and translation of Sptbn2 mRNA, promoting neuronal differentiation [23]. Conversely, in cardiomyocytes, m7G methylation of ATF5 mRNA increases ATF5 expression, which inhibits proliferation and regeneration [24].

Function in tRNA: The m7G modification at the G46 position of the tRNA variable loop is critical for tRNA stability, accurate codon recognition, and function. In mouse embryonic stem cells (mESCs), METTL1/WDR4 mediates m7G on 22 tRNAs at a “RAGGU” motif, revealing a more extensive tRNA methylome in mammals than in yeast; this modification is essential for mRNA translation, ESC self-renewal and differentiation [25]. In vivo, METTL1/WDR4 dysfunction reduces m7G-tRNA abundance, selectively inhibiting the translation of mRNAs involved in cytoskeleton organization and Rho GTPase signaling. This can result in primordial dwarfism, underscoring its role in development [26]. Furthermore, METTL1-mediated tRNA stability prevents premature senescence. Age-related declines in METTL1/WDR4 expression and m7G-tRNA levels lead to ribosome pausing at specific codons, disrupting proteome homeostasis and activating integrated stress responses [27]. tRNAs can be cleaved into tRNA-derived small RNAs (tsRNAs), which are regulatory non-coding RNAs rather than mere degradation products [28]. These tsRNAs can also bear m7G modifications. Under stresses such as heat shock or nutrient deprivation, cells form cytoplasmic SGs to protect mRNAs [29]. In cadmium-induced stress, METTL1 upregulates translation of the core SG protein G3BP1 via tRNA m7G methylation and enhances the expression of an m7G-modified tsRNA (tsRNA-3′-tiRNA Met^CAT^), thereby influencing SG assembly and highlighting METTL1’s role in stress adaptation [30]. Additionally, the METTL1/m7G/mt-tRF3b-LeuTAA axis regulates mitophagy and metabolic reprogramming, influencing processes such as cartilage degradation in osteoarthritis [31].

Function in miRNA and circRNA: METTL1-mediated m7G also regulates non-coding RNAs (ncRNAs). In the miRNA let-7e-5p, m7G methylation at the G11 site antagonizes G-quadruplex structures in the pri-miRNA, facilitating its processing and maturation [32]. Similarly, METTL1 methylates pri-miR-760 in an m7G-dependent manner, promoting its maturation to miR-760, which targets ATF3 mRNA and promotes tumor proliferation and metastasis [33]. Furthermore, m7G modification is present on circular RNAs (circRNAs). A study identified 937 METTL1-catalyzed m7G-modified circRNAs, predominantly enriched in GG-rich or GT-rich motifs, with most deriving from exons on chromosomes 1 and 2 [34]. In hepatocellular carcinoma, METTL1-mediated m7G sites within circIPP2A2 (+545–683 region) regulates its stability and expression, promoting malignant behaviors [35].

Collectively, these findings demonstrate that the METTL1/WDR4 complex, through its versatile m7G-modifying activity, plays diverse and critical roles across RNA species, providing a fundamental basis for understanding its functions in pathologies such as colorectal cancer.

#### 2.1.2. RNMT/RAM

In eukaryotes, most mRNA translation is initiated via a cap-dependent mechanism, wherein the m7G cap, a conserved 5′ modification installed co-transcriptionally, plays a central role. The RNA guanine-7 methyltransferase (RNMT), in complex with its activating subunit RAM, specifically catalyzes this methylation. RAM stabilizes RNMT and optimizes its active site conformation, thereby promoting efficient substrate binding [36].

The m7G cap protects nascent transcripts from exonucleolytic degradation and recruits cap-binding complexes that mediate pre-mRNA processing, nuclear export, and translation initiation [37]. Cap formation (cap 0, m7GpppN) is a coordinated enzymatic process involving RNA triphosphatase, guanylyltransferase, and methyltransferase activities. Further 2’-O-methyltransferases by CMTR1 and CMTR2 produce cap 1 (m7GpppN1m) and cap 2 (m7GpppN1mpN2m), respectively. These modifications are critical for distinguishing endogenous RNA from foreign nucleic acids, thereby suppressing aberrant innate immune activation, and are essential for embryonic development [38]. RNMT activity is precisely regulated: CDK1-cyclin B1 phosphorylation enhances its methyltransferase activity during the G2/M phase, ensuring a surge of cap methylation as cells exit mitosis, a rate-limiting step for proliferation [39]; c-Myc upregulates m7G cap formation by increasing RNMT recruitment to transcription sites, highlighting the cap’s crucial role in Myc-driven gene expression, protein synthesis, and cell growth [40]. TOP (terminal oligopyrimidine) mRNAs are a functionally important class regulated by RNMT; its upregulation—for example, during T cell activation—drives ribosome biogenesis, which is vital for cell growth and differentiation [41]. Thus, RNMT/RAM is not merely a fundamental housekeeping protein but likely functions as a hub regulated by oncogenic signals, integrating translational control and immune modulation.

#### 2.1.3. WBSCR22/TRMT112

Williams–Beuren syndrome chromosomal region 22 (WBSCR22) harbors a canonical SAM-binding motif and forms a stable heterodimer with the tRNA methyltransferase activator subunit 11-2 (TRMT112), which protects it from proteasomal degradation [42]. This complex catalyzes m7G methylation at position G1639 of 18S rRNA, a modification critical for the efficient processing of the nuclear 18S rRNA precursor. Loss of WBSCR22 results in nuclear accumulation of the 18SE pre-rRNA intermediate, impairing 18S rRNA maturation and late pre-40S ribosomal particles export. Intriguingly, its catalytic methyltransferase activity is dispensable for this processing function [43], consistent with a broader paradigm where the physical presence of certain modifying enzymes, rather than their catalytic activity, is a structural prerequisite for accurate pre-rRNA processing [44].

The WBSCR22/TRMT112 complex is evolutionarily conserved. Its functional ortholog in caenorhabditis elegans, BUD23, mediates intergenerational epigenetic regulation: parental starvation modulates 18S rRNA m7G methylation via BUD23, altering ribosomal occupancy on transcripts related to lifespan, stress response, and reproduction, thereby transmitting metabolic information to offspring [45]. Notably, WBSCR22 (also called Merm1) has a role beyond rRNA modification: its N-terminal SAM-binding domain is recruited by Dnmt3a, inhibiting Dnmt3a’s catalytic activity and promoting rRNA transcription [46]. Thus, WBSCR22 functions as a dual-capacity regulator, influencing ribosome assembly via direct rRNA binding and epigenetic modulation of rRNA transcription.

Beyond the canonical complex, additional potential m7G regulators have been identified with diverse subcellular localization and tissue-specific functions (Figure 1B). For example, methyltransferase-like protein 7A (METTL7A) is a mechanosensitive, non-canonical m7G methyltransferase that mediates internal m7G on endothelial mRNAs enriched in a U/A-AG-G/A motif, enhancing the stability of transcripts such as KLF4 and NFKBIA to maintain vascular homeostasis [47]. Similarly, the mitochondrial tRNA methyltransferase TRMT10C has been reported to mediate m7G modification of circFAM126A, enhancing its stability [48], and METTL17 has been implicated in influencing m7G modification of mitochondrial tRNAs, thereby regulating mitochondrial translation and energy metabolism [49]. However, whether these proteins directly catalyze m7G deposition or indirectly influence the modification status warrants rigorous biochemical validation.

### 2.2. Erasers

The reversible regulation of RNA modifications has been established for certain marks, such as m6A, whose methyl group can be efficiently removed by oxidative demethylases (e.g., ALKBH5 and FTO), enabling precise control of gene expression [50]. In contrast, the chemical nature of m7G, a quaternary ammonium structure, confers high chemical stability, making it resistant to known oxidative demethylation pathways. Whether m7G modifications are dynamically regulated, and if dedicated eraser enzymes exist, remains an open question. Although the m6A eraser ALKBH1 has been reported to reduce m7G levels on specific transcripts such as FZD8 mRNA and promote its decay [51], this observation hints at a potential cross-reactivity rather than establishing a bona fide m7G demethylase, and definitive evidence is still lacking. To date, no enzyme has been conclusively validated as a physiological m7G eraser. Therefore, the hypothetical existence of a novel enzymatic mechanism capable of cleaving the stable methylguanosine bond of m7G represents a major frontier, albeit still a speculative one in epigenetic research.

### 2.3. Readers

Reader proteins execute the functional outcomes of m7G modification by specifically recognizing and binding to methylated sites, thereby regulating critical steps in RNA metabolism such as mRNA export, translation, and quality control.

#### 2.3.1. Internal mRNA Readers: QKI, IGF2BP Family, and GEMIN5

The Quaking (QKI) protein family, particularly the QKI7 isoform, functions as a reader that specifically recognizes internal m7G modifications. QKI7 directly binds to 30–50% of internal m7G sites. Under cellular stress, QKI7 preferentially binds to cytoplasmic mRNAs enriched in GA motifs and interacts with the stress granule core protein G3BP1, facilitating the transport of m7G-modified mRNAs to stress granules independently of the canonical nuclear export pathway. This sequestration regulates mRNA stability and suppresses translation. Furthermore, QKI7 inhibits the Hippo signaling pathway in an m7G-dependent manner, sensitizing cancer cells to chemotherapeutic agents [21]. Members of the IGF2BP family (IGF2BP1-3) have also been characterized as readers for METTL1-mediated internal m7G. Notably, IGF2BP3 exhibits a preference for binding m7G modifications within the 3′ UTR of mRNAs, where it promotes the degradation of target transcripts [22]. In addition, GEM nuclear organelle-associated protein 5 (GEMIN5), a central regulator of small nuclear ribonucleoprotein particles (snRNPs) assembly, recognizes and binds to internal m7G sites in mRNAs, thereby regulating their stability [52].

**Figure 2:**
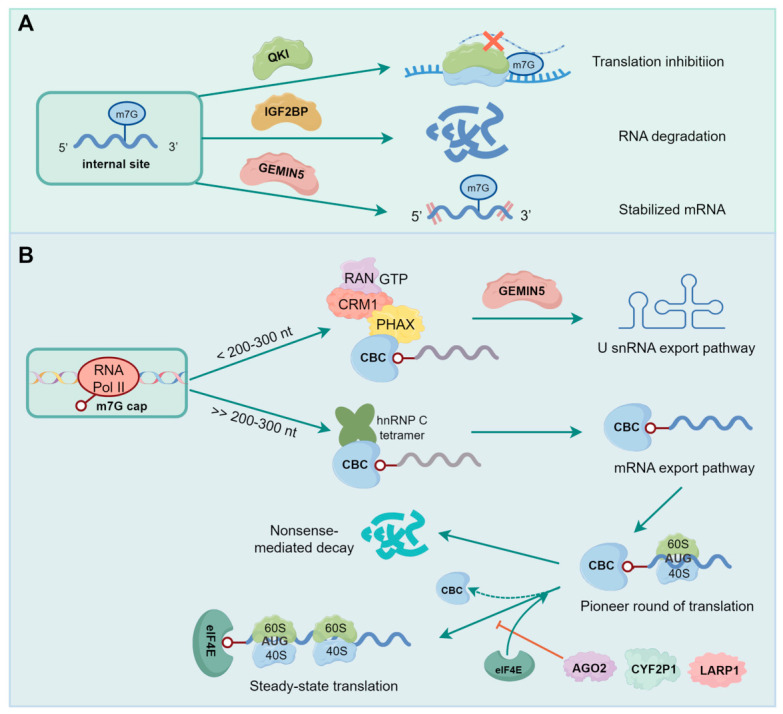
Reader proteins of internal m7G and cap m7G modifications on mRNA. (Created with Figdraw; Export ID: IWPUT32b43, www.figdraw.com (accessed on 6 May 2026).) (**A**) The left panel depicts the reader proteins that recognize mRNA m7G modifications located at internal sites, including QKI, IGF2BP family and GEMIN5. The right panel demonstrates the critical cytological functions on mRNA export, translation, and quality control. (**B**) Reader proteins that recognize mRNA m7G modifications located at the m7G cap, including CBC, eIF4E, Gemin5, AGO2, CYFIP1 and LARP1. CBC determines the fate of RNA export by recruiting distinct effectors depending on the length of the transcripts. CBC also mediates the pioneer round of translation to achieve mRNA quality control. Then CBC is replaced (shown in dashed lines) by eIF4E (shown in solid lines) for steady-state cytoplasmic translation. AGO2, CYFIP1 and LARP1 can competitively bind the m7G cap to repress translation initiation (the inhibitory effect is shown as a red line).

These internal m7G readers profoundly influence mRNA metabolic fate through a distinct molecular mechanism (Figure 2A), and their dysregulation plays a significant role in the pathogenesis of various diseases, including colorectal cancer.

#### 2.3.2. The m7G Cap Readers: CBC, eIF4E, Gemin5, AGO2, CYFIP1 and LARP1

Specific recognition of the m7G cap determines the fate of nascent transcripts. In mammalian cells, cap-dependent translation is mediated by two primary cap-binding complexes: the nuclear cap-binding complex (CBC) and the cytoplasmic eukaryotic translation initiation factor 4E (eIF4E) (Figure 2B).

The CBC, a heterodimer of CBP20 (NCBP2) and CBP80 (NCBP1), is the initial cap binder. CBP20 directly engages the m7G cap, while CBP80 stabilizes this interaction and serves as a scaffold for downstream effectors. The CBC is integral to transcription elongation, nuclear export, and the pioneer round of translation that includes nonsense-mediated mRNA decay (NMD), a crucial quality control mechanism [53]. The m7G cap acts as a molecular switch; the effectors recruited by CBC determine the export pathway. For example, CBC interaction with hnRNP C directs longer transcripts (>200–300 nt) into the mRNA export pathway [54], whereas CBC together with phosphorylated PHAX and CRM1-RanGTP exports shorter capped RNAs (<200–300 nt) into the snRNA pathway for spliceosome assembly [55]. Gemin5 binds the m7G cap of pre-snRNAs via its intact WD-repeat domains [56] and plays a critical role in their cytoplasmic quality control and maturation [57].

Following the pioneer round, CBC is replaced by eIF4E for steady-state cytoplasmic translation [58]. eIF4E, the core cytoplasmic cap-binding protein and a rate-limiting factor for translation initiation, forms the eIF4F complex with eIF4G and eIF4A to recognize and directly bind to the m7G cap of target mRNAs [59]. eIF4G recruits eIF4A, whose helicase activity unwinds mRNA secondary structures to facilitate 40S ribosomal subunit scanning. eIF4G also bridges eIF4E with poly(A)-binding protein (PABP) at the 3′ tail, promoting mRNA circularization, which synergistically enhances translation initiation and stability [60,61]. Furthermore, eIF4E localizes to the nucleus via an importin 8-mediated import [62]. Recent studies show that within the nucleus, eIF4E competitively binds the m7G cap of nascent mRNA against NCBP2, establishing an early “checkpoint” that determines mRNA fate: NCBP2-bound mRNAs are typically processed correctly and exported for translation, whereas eIF4E-bound mRNAs are preferentially channeled into nuclear degradation pathways [63].

Other proteins also competitively bind the m7G cap to regulate translation. For example, AGO2 (via its MC domain) and CYFIP1 form alternative complexes that repress translation initiation [64,65]. A key specialized regulator is La-related protein 1 (LARP1), which through its C-terminal DM15 domain binds with high specificity and affinity to both the m7G cap and the adjacent 5′ TOP motif of TOP mRNAs, thereby outcompeting eIF4E and preventing eIF4F complex assembly on these transcripts. Thus, LARP1 acts as a potent translational suppressor of TOP mRNAs [66]. Functionally, LARP1 buffers cellular protein synthesis capacity: under mTOR-inactive conditions, LARP1 binds and protects TOP mRNAs from translation; upon mTOR activation, this repression is relieved, allowing for rapid translation of stored TOP mRNAs to restore ribosome biogenesis. This positions LARP1 as a critical effector of mTOR signaling in translational reprogramming [67].

Together, the recognition of the m7G cap constitutes a precise, multi-step cascade of protein interactions that dictates the lifecycle and translational fate of mRNAs. This tightly coordinated regulatory network, spanning nuclear processing, quality control, and cytoplasmic translation, ensures fidelity in gene expression. Dysregulation at any node within this cascade can disrupt cellular translation programs, thereby driving malignant tumor proliferation and adaptation.

### 2.4. Other m7G Cap-Related Enzymes

Beyond initial formation and recognition, the m7G cap undergoes further modification and removal, adding regulatory complexity.

Cap hypermethylation by TGS1: Trimethylguanosine synthase 1 (TGS1) catalyzes the dimethylation of the m7G cap to generate m_2,2,7_G-cap (TMG cap). TGS1 exhibits high affinity for specific transcripts, such as mammalian selenoprotein mRNAs and the resulting TMG cap is no longer efficiently recognized by eIF4E, thereby diverting these transcripts to an alternative translation initiation pathway [68]. In addition to its catalytic domain, unique N-terminal and alpha-helical domains of TGS1 are essential for cap binding and activity. Beyond mRNAs, TGS1 also hypermethylates the caps of snRNAs, snoRNAs, and telomerase RNA during their maturation [69].

Decapping enzymes DCP2 and DXO: Controlled cap removal (decapping) is critical for mRNA turnover. The primary decapping enzyme DCP2 recognizes and hydrolyzes the mature m7G cap, especially on mRNAs harboring a stem-loop structure within the first 10 nucleotides of the 5′ end, releasing m7GDP and generating a 5′-monophosphate RNA that is subsequently degraded by XRN1 [70]. In contrast, the trifunctional DXO/Dom3Z possesses decapping, pyrophosphohydrolase, and exonuclease activities. Because cap-binding proteins like CBP20 and eIF4E protect only methylated m7G caps, DXO selectively targets and degrades improperly capped pre-mRNAs that lack this modification, serving as a quality control before splicing and polyadenylation [71]. In brief, DCP2 promotes the decay of mature capped mRNAs, whereas DXO/Dom3Z eliminates non-m7G-capped transcripts for quality control.

Synergy with other RNA modifications: The m7G cap functions synergistically with adjacent modifications to fine-tune mRNA fate. The first transcribed nucleotide adjacent to the cap critically influences mRNA stability and abundance. A prime example is m6Am, a dynamic modification widely present in the cap of most mRNAs. The methyltransferase PCIF1 catalyzes m6Am formation, which can be demethylated by FTO in an m7G cap-dependent manner. Importantly, m6Am confers resistance to DCP2-mediated decapping, thereby enhancing transcript stability [72]. Furthermore, modifications such as internal m6Am on U2 snRNA (mediated by METTL4) can cooperate with the snRNA’s own m7G cap to regulate alternative splicing, illustrating how cap and internal modifications coordinate complex RNA processing events [73].

## 3. Aberrant m7G Modification in the Pathogenesis and Progression of Colorectal Cancer

As previously established, m7G modification and its regulators are integral to diverse biological processes. Their dysregulation, particularly involving the METTL1/WDR4 complex, is increasingly implicated in the initiation and progression of various cancers. For example, METTL1/WDR4-mediated internal mRNA m7G hypermethylation drives Gefitinib resistance in non-small-cell lung cancer [74], while its epitranscriptomic regulation of TXNDC12 facilitates head and neck squamous cell carcinoma progression via c-Myc signaling [75]. Furthermore, METTL1-mediated tRNA m7G modification promotes proliferation and metastasis in papillary thyroid cancer by upregulating TNF-α [76] and confers radiation resistance in hepatocellular carcinoma by enhancing DNA repair [77]. Given these pleiotropic oncogenic roles across cancer types, this section will focus on the specific contributions of aberrant m7G modification and its associated proteins to the pathogenesis and progression of CRC.

### 3.1. METTL1/WDR4 in CRC

**Figure 3:**
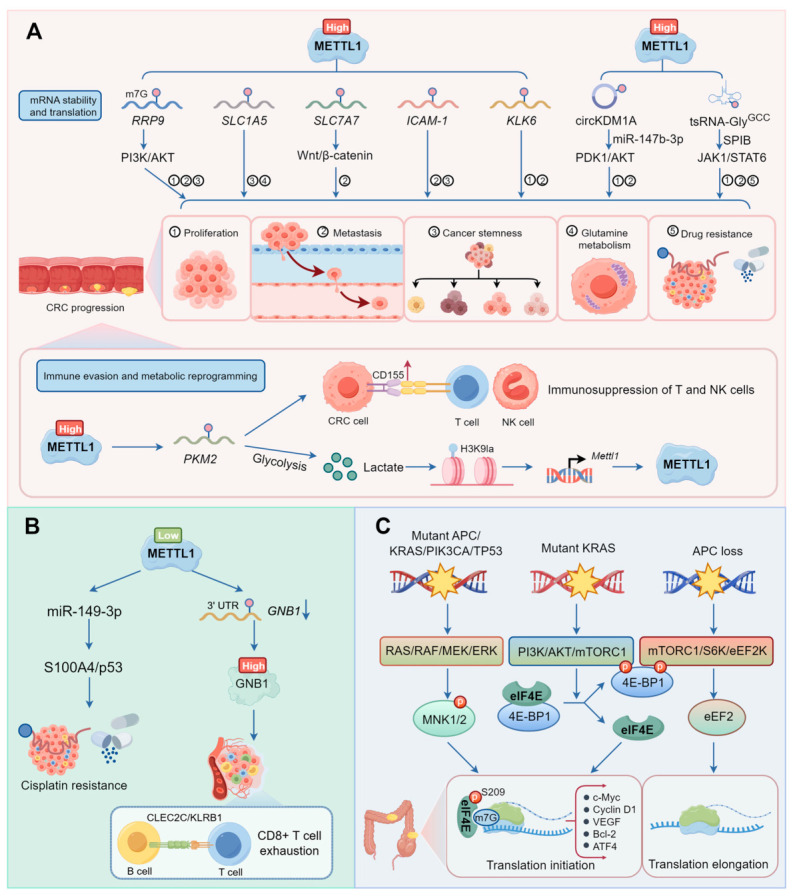
Roles and potential molecular mechanisms of m7G modification in CRC. (Created with Figdraw; Export ID: IITYI55e3e, www.figdraw.com (accessed on 6 May 2026).) (**A**) High expression of METTL1 regulates various tumor processes through m7G modification, including proliferation, metastasis, cancer stemness, metabolism reprogramming, drug resistance and immune evasion. (**B**) Low expression of METTL1 exerts an important tumor-suppressive function. (**C**) eIF4E functions as a central hub that couples oncogenic signaling to translational dysregulation in CRC.

The m7G methyltransferase METTL1, along with its obligate partner WDR4, plays a critical and multifaceted role in the pathogenesis of CRC (Figure 3A). Its expression and activity are regulated by a complex network of oncogenic signals, profoundly impacting tumor progression through diverse molecular mechanisms.

METTL1 promotes CRC cell proliferation by alleviating cell cycle arrest. It suppresses CHEK2 expression, thereby attenuating G1/S phase arrest and facilitating uncontrolled proliferation [78]. Multiple independent cohorts and in vitro loss-of-function experiments consistently show that METTL1-driven internal mRNA m7G modifications in CRC promote tumor progression by stabilizing key oncogenic transcripts and activating downstream signaling pathways. Mechanistically, METTL1 stabilizes specific mRNAs to activate pro-tumorigenic pathways: it stabilizes RRP9 mRNA to activate PI3K/AKT signaling [79], stabilizes ICAM-1 mRNA to boost invasion, migration, and stemness [80], and cooperates with the RNA-binding protein QKI to stabilize SLC7A7 mRNA, activating Wnt/β-catenin to drive metastasis [81]. Furthermore, METTL1 collaborates with upstream regulators through m7G modification to drive colorectal cancer progression. CAF-derived exosomes facilitate glutamine metabolism and tumor stemness via METTL1-mediated m7G modification of SLC1A5 mRNA [82], while hsa_circ_0003323 binds to METTL1 to induce m7G methylation of KLK6 mRNA, enhancing its stability and thereby promoting CRC proliferation and metastasis [83].

METTL1-mediated tRNA m7G modification drives oncogenic transformation through codon-biased translation [84] or reduced ribosome pausing on cancer-promoting transcripts [85]. Upregulated in CRC and CRC liver metastasis (CRLM), METTL1 correlates with poor prognosis and promotes proliferation, invasion, and metastasis by selectively enhancing translation of oncogenic transcripts, particularly those involved in cell cycle regulation (e.g., CCND3) and PI3K/AKT signaling [86]. Beyond tRNA modification, METTL1 facilitates the biogenesis of specific tRNA-derived small RNAs (tsRNAs). m7G methylation of tRNA-Gly^Gcc^ produces tsRNA-Gly^Gcc^, which promotes proliferation, migration, and 5-fluorouracil resistance via SPIB and the JAK1/STAT6 pathway [87]. METTL1 also modifies circular RNAs, stabilizing circKDM1A through an m7G-modified GG motif; circKDM1A then sponges miR-147b-3p to upregulate PDK1 and activate the AKT pathway [34].

Beyond promoting tumor cell intrinsic malignancy, METTL1 critically shapes the tumor immune microenvironment to facilitate immune evasion. It upregulates PKM2 expression, increasing glycolysis and lactate production; lactate, in turn, promotes histone H3K9 lactylation, which further upregulates METTL1 in a self-reinforcing loop [88]. Nuclear PKM2 dimers promote CD155 expression, inducing immune escape. In liver metastasis, METTL1 knockdown reduces m7G modification in the 3′ UTR of GNB1 mRNA, paradoxically increasing GNB1 expression, which then fosters an immunosuppressive microenvironment by exhausting CD8^+^ T cells via the CELC2C–KLRB1 axis, ultimately promoting metastasis [89]. The tumor microenvironment also modulates METTL1: hypoxia upregulates HIF-1α, which transcriptionally represses METTL1, leading to a more pronounced decrease in tRNA m7G modification compared to mRNA [90].

WDR4, as METTL1’s essential partner, exhibits parallel oncogenic functions. High WDR4 expression correlates with poor prognosis in CRC. It promotes proliferation, migration, and invasion by activating the GSK3β/β-catenin pathway [91]. Its expression can be activated by the P300/ETV4 complex. Subsequently, WDR4 stabilizes SPP1 mRNA via m7G modification, promoting cell cycle progression and suppressing autophagy [92]. This regulatory axis (P300/ETV4/WDR4/SPP1) highlights a conserved mechanism, as a similar P300/SP1 complex transcriptionally upregulates METTL1 to stabilize CDK14 mRNA in prostate cancer [93].

Collectively, these studies establish METTL1/WDR4 as a pivotal oncogenic complex in CRC. However, some contradictions warrant further attention. While most studies report an oncogenic role for METTL1, a paradoxical chemo-sensitizing role has emerged in specific CRC types and drug-resistant microenvironments, where METTL1 overexpression increased apoptosis and sensitivity to cisplatin in colon cancer cells by regulating the miR-149-3p/S100A4/p53 axis (Figure 3B) [94]. Downregulation of METTL1 may be a tumor-evolved mechanism for chemotherapy evasion, revealing a novel tumor-suppressive function via regulation of the non-coding RNA miR-149-3p. This duality provides a new perspective for understanding the role of METTL1 in tumor chemotherapy resistance. Such discrepancies may arise from cell-type specificity, differential microenvironments, or off-target effects, calling for systematic side-by-side comparisons. In addition, the roles of METTL1 in internal m7G modification versus miRNA processing are inconsistently addressed, confounding mechanistic interpretation. Overall, while the pro-tumorigenic role of METTL1/WDR4 is well-supported, discrepancies regarding subtype-specific effects and molecular targets highlight the need for standardized experimental models and direct comparative studies.

### 3.2. eIF4E in CRC

Although METTL1/WDR4 is a major driver, other m7G-associated proteins also play critical roles in specific contexts and should not be overlooked. eIF4E, the canonical reader of the m7G cap, serves as a central hub linking oncogenic signaling to dysregulated translation through its aberrant expression and activity (Figure 3C). As a key oncogene, eIF4E is frequently overexpressed in approximately 30% of CRC. Its expression level increases progressively during the transition from benign dysplasia to adenocarcinoma [95]. eIF4E overexpression is associated with an increased risk of liver metastasis, advanced clinical stage, and poor prognosis [96,97].

In normal cells, eIF4E activity is tightly regulated to ensure translational fidelity and cellular homeostasis. It is primarily controlled by the PI3K/AKT/mTOR oncogenic pathway, which phosphorylates 4E-BP1, causing its dissociation from eIF4E. This releases eIF4E to assemble the eIF4F initiation complex, thereby integrating growth factor, energy, and nutrient signals to drive the anabolic program of the cell [98]. In contrast, the RAS/RAF/MEK/ERK pathway phosphorylates eIF4E at Ser209 via MNK1/2 kinases. This phosphorylation enhances the association of eIF4E with eIF4G and promotes the translation of specific mRNAs without directly affecting its cap-binding ability [99]. Rather than uniformly enhancing global translation, eIF4E preferentially upregulates specific mRNAs with complex 5′-UTRs that code for key oncogenic factors, including c-Myc, cyclin D1, VEGF, and Bcl-2, thereby promoting malignant phenotypes [100]. Traditionally, mTORC1 is understood to drive oncogenesis via 4E-BP1-mediated control of translation initiation. This is exemplified in PIK3CA-mutant colorectal cancer, where hyperactive PI3K/Akt/mTORC1 signaling upregulates the translation of oncogenic factors like c-Myc and Cyclin D1 [101]. By contrast, in APC-deficient intestinal tumors, mTORC1 promotes protein synthesis via the S6K/eEF2K/eEF2 axis to accelerate translation elongation, which becomes the rate-limiting step in protein synthesis following APC loss [102]. Mutant KRAS elevates p-eIF4E levels by activating the ERK/Mnk pathway. Phosphorylation of eIF4E at Ser209 enhances the translation of pro-tumorigenic proteins such as c-Myc and ATF4, thereby activating the integrated stress response and glutamine metabolism to drive tumor progression [99]. Key CRC driver mutations (APC, KRAS, SMAD4, TP53) synergistically amplify global protein synthesis via the eIF4E-4E-BP1 axis, making it a central hub for oncogene-driven translational reprogramming. Consequently, advanced multi-mutant CRC shows heightened sensitivity to translational suppression, revealing a shared vulnerability to eIF4E-dependent regulation across heterogeneous genetic backgrounds [103].

Therefore, targeting eIF4E or its regulatory network represents a promising precision therapeutic strategy for advanced, multi-mutant colorectal cancer. Rather than merely inhibiting a single pathway, this approach aims to disrupt a central signaling and translational control hub, thereby potentially overcoming treatment resistance caused by pathway redundancy or bypass activation.

### 3.3. Other Regulatory Proteins in CRC

Beyond METTL1/WDR4 and eIF4E, several other m7G-associated regulators play critical roles in CRC progression through diverse mechanisms.

The m7G methyltransferase WBSCR22 is highly expressed in CRC and correlates with poor prognosis. Its suppression not only impairs cancer stem cell properties, proliferation, and invasion [104] but also sensitizes cells to oxaliplatin by promoting apoptosis and reactive oxygen species production [105]. Notably, natural killer cells can counteract oxaliplatin resistance by downregulating WBSCR22 via microRNA-146b-5p [106]. Furthermore, the circular RNA circWBSCR22, derived from WBSCR22 pre-mRNA back-splicing, is upregulated in CRC tissues and promotes epithelial–mesenchymal transition and metastasis [107].

In contrast, reduced expression of the RNA-binding protein QKI is associated with higher tumor recurrence and poorer survival in CRC patients [108]. Mechanistically, QKI deficiency attenuates PABPN1-mediated liquid–liquid phase separation, leading to increased usage of proximal poly(A) sites and ultimately enhancing cancer cell proliferation and migration [109]. Within the nucleus, the cap-binding complex component CBP20 recognizes the m7G cap and regulates the export and translation of mRNAs encoding proteins such as cyclins and growth factors. Its elevated expression in CRC is linked to unfavorable survival, and CBP20 knockdown suppresses tumor growth, highlighting its therapeutic potential [110]. At the translational level, LARP1 and the oncoprotein MYC establish a positive feedback loop. MYC transcriptionally activates LARP1, which then binds the 5′ UTR of MYC mRNA to enhance its translation. This reciprocal activation sustains high expression of both proteins, thereby accelerating CRC progression [111]. Beyond its role in snRNP assembly, GEMIN5 promotes tumorigenesis by increasing m7G modification levels on SHMT2 mRNA, thereby stabilizing it. The resulting GEMIN5–SHMT2 axis inhibits ferroptosis in CRC cells and activates the Wnt/β-catenin signaling pathway [52].

To summarize, m7G-mediated regulation in CRC constitutes an extensive network that goes beyond conventional translation initiation. This network integrates writers, readers, and effector proteins, intersecting with broader RNA processing mechanisms. Rather than focusing solely on METTL1, it is the convergence of these multiple m7G regulators on common pathways that coordinately regulates key oncogenic programs, including metabolic reprogramming, apoptosis evasion, stemness maintenance, and metastasis, through enhanced translation, mRNA stabilization, and altered RNA processing. Therefore, targeting key nodes within this expanded m7G regulatory axis represents a potential multi-faceted therapeutic strategy for colorectal cancer.

## 4. m7G Modification in the Prognosis and Diagnosis of Colorectal Cancer

Despite being a cornerstone of clinical management, the traditional TNM staging system for CRC often fails to capture heterogeneity in patient outcomes, underscoring the critical need for more precise, molecularly defined prognostic biomarkers. In this context, m7G modification and its regulatory network have been proposed as a promising source of such biomarkers, potentially complementing conventional staging. However, an in-depth review of the available evidence reveals considerable gaps that limit clinical translation, a central disconnect between preclinical findings and clinical application.

Multiple studies have used transcriptomic data to build prognostic models based on m7G-related genes. A seven-gene-pair signature (including NCBP2 and DXO) developed using TCGA (571 cases) and GEO (403 cases) could predict overall survival and correlate with tumor mutational burden, microsatellite instability, and immunotherapy response, showing reasonable external validation, but mechanistic validation is lacking [112]. Another four-gene signature (HSF4, ZNF767P, UPK3B, AGAP9) was claimed to predict survival in colon adenocarcinoma and was validated in dual cohorts (TCGA: 480; GEO: 566) and confirmed by qRT-PCR, yet direct m7G evidence and added clinical value remain unverified [113]. A pan-cancer model (TCGA: about 600 cases) has suggested that m7G regulators are conserved across cancers, with widespread overexpression associated with poorer outcomes and reduced immunotherapy efficacy, but this lacks external validation, and clinical utility has not been quantified [114]. Beyond survival prediction, a 15-gene prognostic score (e.g., AGO2, CYFIP1, EIF4E, METTL1) was validated in TCGA (456) and two GEO cohorts (566 + 177) with dual external validation and could be used to stratify patients into risk groups with differential sensitivities to agents like gefitinib, cisplatin, erlotinib, and vinblastine, yet incremental clinical benefit remains unquantified [115]. Non-coding RNAs include a 7-miRNA signature (TCGA: 442 cases), without external validation, for early intervention by identifying high-risk with poor survival [116]; a 17-lncRNA model (TCGA: 647 cases) built to distinguish immunologically “hot” (immune-inflamed) from immunologically “cold” (immune-desert) tumors for immunotherapy stratification, with a marked AUC decline from training to test sets and no external validation [117,118]; and an RNA methylation-related signature (RMS) integrating m6A/m5C/m1A/m7G (TCGA: 600; GEO: 389), yielding AUC values of 0.603–0.891 and a higher C-index (average: 0.722) than 34 previously published models. Although claimed to be superior to single-modification models, the RMS lacks a head-to-head comparison against clinical benchmarks [119].

m7G markers also show potential for early diagnosis and risk assessment. Given the elevated CRC risk in patients with ulcerative colitis (UC) [120], five m7G-related genes (including QKI) were implicated in inflammation and neoplastic transformation [121]. For mucinous adenocarcinoma (MC), a six-lncRNA model (TCGA: 72 MC cases) reported internal AUCs of 0.944–1.000 (suggesting overfitting), with no external validation and small sample size [122]. The field is rapidly advancing toward minimally invasive liquid biopsy applications [123]. m7G-modified nucleosides, detectable in biofluids through robust analytical methods, including UPLC-MS/MS, may pave the way for their application in CRC detection [124]. Twelve m7G-targeted serum miRNAs have demonstrated high pan-cancer accuracy (including CRC) but lack CRC-specific validation, requiring independent cohort and subgroup analyses [125].

Collectively, despite the demonstrated potential of m7G-related markers for prognosis and diagnosis in colorectal cancer, current studies commonly suffer from limitations such as a lack of external validation, failure to quantify the incremental clinical utility over existing biomarkers, and an absence of direct evidence for m7G modification. Clinical translation urgently requires prospective multi-center validation and experimental support with direct detection of m7G modification.

## 5. Therapeutic Potential of Targeting m7G Modification in CRC

As comprehensively discussed, m7G modification plays a pivotal role across the spectrum of CRC, from pathogenesis and progression to prognosis and diagnosis. This central involvement underscores m7G-related proteins and molecules as a promising new class of therapeutic targets for intervention.

### 5.1. Targeting METTL1/WDR4

Although direct studies of METTL1/WDR4 inhibitors in CRC are still rare, pre-clinical evidence points to their significant therapeutic potential. Targeting METTL1 has shown promise in modulating both chemotherapy response [87] and anti-tumor immunity [88]. However, to date, all METTL1/WDR4-targeting strategies remain exclusively at the preclinical proof-of-concept stage, with no agent having entered clinical trials for CRC.

Notably, the first small-molecule inhibitors of METTL1 have been discovered through integrated high-throughput fragment docking and biochemical screening. These adenine derivatives act as competitive inhibitors of the essential co-substrate SAM at the METTL1 catalytic site. Among them, compound 6 is the most potent METTL1 inhibitor in this study, exhibiting good selectivity and a stable predicted binding mode. However, its lack of experimental co-crystal structures, cellular activity, and ADME data restrict it to a hit-to-lead starting point, far from clinical candidacy [126]; thus, it represents a viable starting point for drug development in CRC, but it has not yet been validated in any in vivo efficacy or toxicity model. The application of METTL1 inhibitors may be particularly impactful in specific contexts. During early CRC tumorigenesis, the stepwise progression from normal tissue through dysplasia to cancer was paralleled by a shift in tumor-associated macrophages from a pro-inflammatory to an immune-suppressive phenotype [127]. Given that METTL1-driven m7G methylation of Sarm1 mRNA was known to promote pro-inflammatory macrophage polarization [128], a METTL1 inhibitor like SA91-0178, identified via virtual screening, could potentially reverse this deleterious polarization in the early tumor niche, reducing immune-suppressive infiltration and exerting a therapeutic effect at the precancerous or early cancer stage. Nevertheless, this hypothesis remains strictly preclinical and requires in vivo validation.

**Figure 4:**
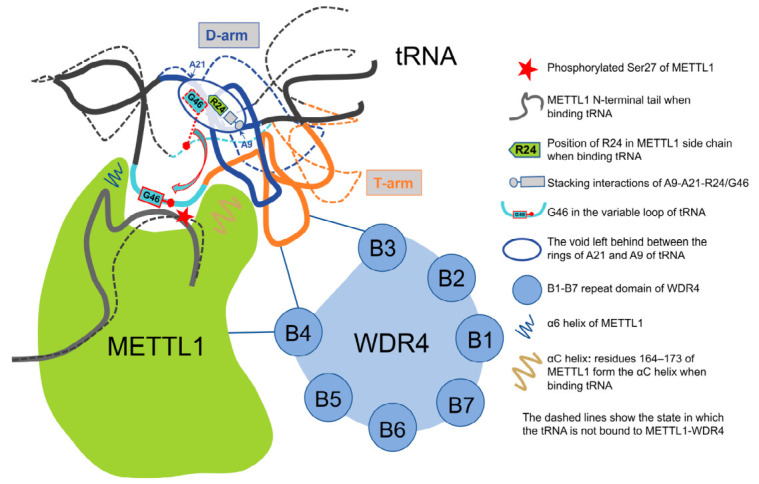
Mechanism of METTL1-WDR4 complex in regulating tRNA m7G modification (Adapted from references [129,130]). The solid-line portion in the upper part depicts METTL1 (green) with its N-terminal tail, αC and α6 helices, along with the B3–B4 region of WDR4 (blue), mediating tRNA m7G methylation at G46. The dashed-line portion shows the state where tRNA is not bound to the METTL1–WDR4 complex.

A major advancement in this field is the elucidation of high-resolution structures of the METTL1-WDR4 complex in complex with tRNA substrates. These structural studies revealed the precise molecular choreography of m7G deposition: WDR4, with its classic WD-repeat propeller structure, uses its B3–B4 domains to dock both METTL1 and the tRNA backbone. METTL1 undergoes a conformational change, with its αC region forming a helix that, along with the α6 helix, anchors the tRNA variable loop. A key arginine residue (R24) displaces the target guanosine (G46), occupying the space between bases A9 and A21, thereby activating the tRNA and flipping G46 into the catalytic pocket for methylation. Furthermore, a regulatory mechanism involving phosphorylation at the N-terminus of METTL1’s S27 residue has been identified, which can allosterically inhibit catalytic activity via steric hindrance. In addition, the binding of tRNA to the METTL1–WDR4 complex is mediated predominantly through interactions with the phosphate groups of the tRNA backbone and does not require direct contact with internal nucleobases. Moreover, the presence of the RGG46UY sequence within the variable loop region of the tRNA substantially enhanced overall m7G methylation efficiency (Figure 4) [129,130]. These atomic-level insights are invaluable, as they map critical functional residues and interaction interfaces, offering hope for the structure-based design of next-generation, highly specific inhibitors that can disrupt this oncogenic complex. However, translation to clinically useful METTL1 inhibitors will require years of iterative medicinal chemistry and rigorous in vivo safety assessment.

Overall, all METTL1/WDR4-directed strategies are strictly preclinical; no inhibitor has reached clinical validation. While high-resolution structures provide a rational template for future design, substantial optimization and safety evaluation are required before any METTL1 inhibitor can enter CRC clinical trials.

### 5.2. Targeting eIF4E

As a critical integrator of multiple oncogenic signals and a master regulator of global and selective protein synthesis, eIF4E plays a pivotal role in the initiation, progression, and therapy resistance of CRC. Clinical exploration, such as a trial combining the eIF4E antisense oligonucleotide (ISIS 183750) in irinotecan-resistant CRC patients, demonstrated disease stabilization in a subset of patients, though it did not meet its primary endpoint [131]. Given its central role, therapeutic strategies targeting eIF4E and its regulatory network represent a major research focus.

Multiple strategies have been developed to directly target eIF4E, although direct strategies remain exclusively preclinical and encounter significant pharmacological and safety barriers that, as yet, prohibit clinical translation. Small-molecule cap-binding competitors, such as 4EGI-1, disrupt eIF4E-eIF4G interaction and inhibit eIF4F complex assembly, showing preclinical synergy with BRAF inhibitors [132]. To address the poor cell permeability of traditional cap analogs, an acyclic nucleoside phosphonate prodrug-based strategy has been employed to develop novel cell-permeable eIF4E inhibitors [133], while computational studies suggest the polysaccharide sizofiran as a potential inhibitor [134]. Both remain in the early discovery stages with no in vivo validation of target engagement. Beyond de novo drug design, drug repurposing offers another direct strategy: the antiviral agent ribavirin acts as a functional mimetic of eIF4E, competitively inhibiting its activity. In preclinical CRC models, ribavirin suppresses proliferation, induces apoptosis, and reverses chemoresistance [135]. Its mechanism may further involve the downregulation of PRMT5 and associated histone modifications (H3R8me2s, H4R3me2s), thereby reducing eIF4E expression [136]. Despite ribavirin’s approval for other indications, its use as a specific eIF4E inhibitor in CRC remains preclinical, and its antitumor effects are confounded by off-target activities. Advancements in research methodologies have further propelled this field. capCLIP enables genome-wide mapping of the eIF4E-mRNA interactome, facilitating the discovery of oncogenic translation programs [100]. Meanwhile, the CRISPR RiPCA platform allows for in situ, dynamic, and quantitative analysis of eIF4E–m7G cap interactions in living cells, providing a powerful tool for high-throughput drug screening [137]. However, neither approach obviates the fundamental challenges inherent to direct targeting strategies.

Beyond direct targeting, eIF4E activity can also be modulated indirectly through its upstream regulators, which are more advanced but still have issues. Inhibitors of the upstream kinase MNK, such as eFT508 and ETC-206, exert antitumor effects primarily by reducing phosphorylated eIF4E (S209) levels. Despite being the most clinically advanced eIF4E-modulating strategies, neither ETC-206 in phase II for metastatic CRC nor eFT508 completed in MSS relapsed/refractory CRC has shown breakthrough monotherapy or combination efficacy in colorectal cancer, underscoring an urgent need for structurally distinct MNK inhibitors [138]. In addition to MNK, the upstream kinase mTOR represents another key target. Both mTOR inhibitors (e.g., rapalogs) and dual PI3K/mTOR inhibitors (e.g., NVP-BEZ235) have been shown to suppress CRC cell proliferation and reduce 4E-BP1 phosphorylation in preclinical models [139]. However, mTOR inhibition often triggers feedback resistance, prompting alternative strategies such as reactivation of the B56-PP2A complex to dephosphorylate and reactivate 4E-BP, thereby inhibiting oncogenic translation [140]. Other agents (metformin [141], aspirin [101]) act through broader signaling networks to converge on eIF4E regulation, but these are repurposed drugs with pleiotropic effects, not validated on-target eIF4E inhibitors. Given the limitations of monotherapy targeting eIF4E or a single upstream pathway, which often results in limited efficacy or resistance, current evidence points to the potential superiority of MNK inhibitors and eIF4E/eIF4G interaction inhibitors over mTOR inhibitors. Combined approaches targeting MNK and the eIF4E/eIF4G interface hold theoretical promise, but their clinical feasibility awaits demonstration of a favorable therapeutic index [142].

Taken together, eIF4E is a compelling therapeutic target in CRC. Direct and indirect inhibition strategies have been developed, with combination approaches targeting MNK and eIF4E/eIF4G showing promise in overcoming resistance and improving efficacy; however, further investigation is required.

### 5.3. Other Therapeutic Strategies

In addition to the well-characterized roles of METTL1/WDR4 and eIF4E in CRC pathogenesis and therapy, other m7G-associated proteins and technological applications offer further dimensions for therapeutic intervention.

The therapeutic strategy extends beyond the writers to their critical downstream effectors. A critical example is GNB1, whose mRNA m7G methylation was mediated by METTL1, driving CRCLM. Intriguingly, CRLM patients with high GNB1 expression showed greater benefit from immunotherapy, positioning GNB1 not only as a promoter of metastasis but also as a predictive biomarker and a promising co-target for personalized combination therapies aimed at enhancing immunotherapy efficacy [89]. The methyltransferase WBSCR22 emerges as a compelling target for overcoming chemotherapy resistance. Its suppression not only impairs core oncogenic phenotypes but also re-sensitizes cells to oxaliplatin. Pharmacological inhibition of WBSCR22 could be synergistic with standard chemotherapies [105]. The RNA-binding protein QKI represents a target for restoration therapy. Strategies aimed at restoring or mimicking QKI function could suppress the pro-migratory phenotype associated with its deficiency. This approach targets a post-transcriptional vulnerability distinct from conventional pathways [108]. The CBP20, with its direct role in exporting oncogenic mRNAs, is a prime candidate for interception therapy. Inhibiting CBP20’s cap-binding activity could selectively disrupt the nuclear export and translation of key cancer-driving transcripts [110]. The LARP1-MYC positive feedback loop presents a classic oncogenic dependency. Therapeutic disruption of this loop could break the self-reinforcing cycle that maintains high MYC levels. This is particularly attractive given the historical difficulty faced in directly targeting the MYC transcription factor itself [111]. Finally, the GEMIN5-SHMT2 axis connects m7G modification to metabolic reprogramming and cell death resistance. Inhibiting GEMIN5 could sensitize CRC cells to ferroptosis, a regulated cell death process with growing therapeutic interest. Concurrently, disrupting this axis provides a dual therapeutic effect [52]. These m7G-associated proteins may hold potential promise in the treatment of CRC and are strictly preclinical, requiring further in-depth exploration.

Furthermore, the m7G cap structure is also central to advancing mRNA-based therapeutics. With advantages in ease of design, production, efficacy, safety, and specificity over traditional strategies, mRNA vaccines represent a promising emerging immunotherapy for CRC that can enable precision oncology and shorten the development cycle for personalized treatments [143]. While linear mRNA molecules are susceptible to degradation and can trigger acute immune stimulation, incorporating the m7G cap—either covalently or non-covalently—into circular mRNA platforms significantly enhances transcript stability, translation efficiency, and duration of protein expression. This optimization provides a crucial technical foundation for developing next-generation mRNA vaccines and therapeutic platforms against CRC [144,145]. To date, no mRNA vaccine has yet been approved for CRC, and their therapeutic efficacy remains under clinical investigation.

Collectively, these m7G-related regulators underscore a layer of cancer biology ripe for therapeutic exploitation. Their mechanisms, spanning mRNA modification, stability, export, and translation, converge to sustain hallmark capabilities of CRC. Currently, eIF4E-targeted agents represent the most clinically advanced m7G-related strategy, although their therapeutic window is narrow due to global translation suppression. Targeting METTL1/WDR4 remains at an early preclinical stage (Table 1). No direct m7G writer inhibitor has yet entered clinical trials for CRC, with pharmacologic and safety hurdles remaining to be overcome.

## 6. Challenges and Limitations in m7G Modification

In recent years, the role of m7G modification in CRC has attracted widespread attention; however, related research still faces numerous controversies and technical bottlenecks, including an incomplete regulatory network, a lack of identified demethylases, and limited resolution of detection methods. Here, we synthesize these major debates and limitations to provide a critical reference for future research.

The current understanding of m7G modification in CRC is challenged by several key issues. First, although methyltransferases such as METTL1/WDR4, RNMT/RAM, and WBSCR22/TRMT112 have been identified, the regulatory network remains incomplete, as proteins like METTL7A, TRMT10C, and METTL17 lack direct catalytic evidence, and unique m7G systems may exist in subcellular compartments such as mitochondria. Moreover, no specific demethylase has been found under physiological conditions due to the chemical stability of m7G, limiting insights into its dynamic regulation. In addition, m7G frequently cross-talks with other modifications; for example, m6Am adjacent to the cap forms a critical regulatory module, and FTO-mediated m6Am demethylation affects mRNA stability and cancer stemness [146], while METTL3-dependent m6A upregulates METTL1 to stabilize pro-oncogenic transcripts like CDK4 [147]. Functionally, beyond serving as a signal for conventional translation initiation, m7G exhibits RNA-type- and position-dependent effects: the cap enables non-canonical translation of cryptic transcripts, as seen in C9orf72 repeats [148] and lncRNA-encoded microproteins such as SMIMP [149], whereas internal m7G promotes mRNA degradation via the 3′ UTR and modulates tRNA structure, miRNA processing, and circRNA stability. Collectively, these complexities underscore the need for high-precision methods to decipher the m7G regulatory network in CRC.

Currently, multiple methods are available for detecting m7G, but each has inherent limitations. LC–MS enables quantitative m7G analysis yet lacks throughput and single-nucleotide resolution [150]. Antibody-based approaches, such as MeRIP-seq, enable transcriptome-wide mapping of internal m7G methylation but are resolution-limited (100 bp) [12]; miCLIP-Seq achieves higher resolution (30 bp) [20], yet both are prone to false positives due to non-specific binding. To address this, m7G-MaP-seq and TRAC-seq exploit m7G’s unique chemistry to reduce false positives and enable single-base absolute quantification of internal sites, albeit demanding high-quality RNA and involving laborious procedures [151,152]. For cap-specific analysis, CapQuant enables systematic cap discovery, metabolite cap detection, and absolute quantification, but requires complex, time-consuming preparation and is unsuitable for very low input samples [153]. In contrast, CAP-MAP rapidly and simply detects major m7G cap methylation status, yet it requires high-resolution LC-MS and cannot cover C/U-starting transcripts or non-canonical cap structures [154]. A more recent advance, P(III)-amidite-based stable isotope labeling LC-MS/MS, offers highly sensitive and accurate absolute cap quantification, but the internal standard synthesis is complex and only cap structures initiating with adenosine can be addressed [155]. Additionally, machine learning models (e.g., Moss-m7G [156], MCAMEF-BERT [157], Deep-m7G [158]) for predicting m7G sites in RNA cannot distinguish cap from internal m7G, and relying on existing experimental data for training, have limited ability to uncover novel patterns while risking false positives. Overall, distinguishing cap m7G from internal m7G remains challenging due to the lack of a method that can map both simultaneously. Internal m7G is extremely low in abundance and often masked by ubiquitous cap m7G; the two are chemically identical, so antibodies cannot differentiate them. Moreover, internal signals are prone to contamination or loss during sample preparation.

Despite its complex, context-dependent functions in CRC, m7G research is limited by methodological constraints, necessitating more efficient, robust, and clinically feasible detection platforms. Future efforts should prioritize single-nucleotide resolution, ultra-sensitive techniques capable of distinguishing cap from internal m7G, integrated with multi-omics to fully decipher m7G-governed networks in CRC.

## 7. Conclusions and Perspectives

m7G, as a crucial post-transcriptional RNA modification, plays key regulatory roles in various biological processes including RNA stability, processing, nuclear export, and translation efficiency. In CRC, m7G modification and its core regulatory proteins have been demonstrated to be closely associated with tumor initiation, progression, metastasis, chemotherapy resistance, and immune microenvironment remodeling.

Multiple studies suggest that m7G alterations play a causal driver role in CRC, forming a complex regulatory network involving writers (e.g., METTL1/WDR4), readers (e.g., eIF4E, GEMIN5), and effector proteins. METTL1/WDR4 activates the PI3K/AKT pathway by stabilizing mRNAs or tRNAs, while the Wnt/β-catenin pathway is engaged through various mechanisms: METTL1 via SLC7A7, WDR4 via GSK3β/β-catenin, and GEMIN5 via SHMT2. Meanwhile, eIF4E can be activated by the PI3K/AKT/mTORC1 pathway. Furthermore, positive feedback loops—including LARP1–MYC, PKM2–METTL1 lactylation, and c-Myc–cap m7G—further amplify malignancy. These regulators converge at key signaling hubs (including PI3K/AKT, Wnt, and c-Myc) and on translational reprogramming, synergistically driving tumor progression. However, m7G alterations can also arise as a secondary consequence. The hypoxic tumor microenvironment transcriptionally represses METTL1 via HIF-1α, reducing tRNA m7G modification levels. Likewise, upstream oncogenic pathways (e.g., PI3K/AKT, RAS/ERK) activate eIF4E, indicating that eIF4E activation is a downstream event. Thus, m7G modifications and their regulatory proteins act both as upstream drivers and downstream effectors. These two roles are not mutually exclusive; rather, they form a complex causal network during CRC progression. Future studies integrating single-cell multi-omics and spatial transcriptomics are needed to dissect the direction of causality in specific cellular contexts and disease stages.

m7G demonstrates significant value in CRC prognosis prediction, treatment efficacy evaluation, and therapeutic target development. Prognostic models based on m7G-related molecules may serve as a complement to traditional TNM staging, while m7G-modified circulating miRNAs have shown potential as minimally invasive biomarkers. Targeting key regulators such as METTL1/WDR4 or eIF4E might help restore chemosensitivity or suppress malignant progression. Research on METTL1/WDR4-targeting is in the early stages, whereas strategies targeting eIF4E have been relatively well-developed. Nevertheless, further investigation of these strategies is still required. This will include investigation into factors in direct competition with cap-binding activity, inhibiting upstream regulatory kinases such as MNK and mTOR, drug repurposing, and the development of novel antisense oligonucleotides (ASOs). However, the elucidation of eIF4E’s nuclear functions, such as its competition with NCBP2 leading to nuclear mRNA degradation, suggests a novel therapeutic rationale that disrupts nuclear cap-recognition competition to block oncogene expression at an early post-transcriptional stage [63]. The clinical translation of m7G modification faces multiple bottlenecks. (1) Limitations in detection technology: Current m7G detection methods have multiple limitations and cannot distinguish cap from internal m7G. These limitations, along with complex procedures and sample loss risks, hinder reliable clinical translation. (2) Safety of targeted therapy: m7G is extensively involved in normal physiological processes, and systemic inhibition of its key regulatory proteins may lead to off-target toxicities. Developing highly selective inhibitors or tissue-specific delivery systems is therefore crucial. (3) Complexity of regulatory mechanisms and tumor heterogeneity: Core regulatory factors such as METTL1 can play dual roles, either promoting or suppressing cancer in CRC, which increases the complexity of targeted intervention. Simultaneously, the high degree of heterogeneity in CRC poses a challenge for m7G-based precision subtyping. Therefore, future research needs to integrate single-cell multi-omics and spatial transcriptomics technologies to gain deeper insights into the role of m7G modification in tumor heterogeneity and microenvironment remodeling.

In summary, m7G modification plays an increasingly clear and intricate central role in the initiation, progression, and therapeutic response of colorectal cancer. Despite the numerous challenges that remain in terms of both fundamental mechanisms and clinical translation, continued innovation in detection technologies and deeper investigation into its regulatory networks, functional complexity, and crosstalk with other modifications hold significant promise. Precision diagnostic and targeted therapeutic strategies centered on m7G modification are expected to open new avenues for personalized medicine in CRC, ultimately improving patient prognosis.

## Figures and Tables

**Table 1:** Categorization of potential clinical inhibitors and major strategic directions.

Potential Targets	Drug Candidates	Mechanisms	Translational Status	Refs.
METTL1	— *	Competitively inhibiting SAM binding to METTL1	Preclinical	[126]
	SA91-0178	Blocking METTL1-Sarm1-NAD^+^ signaling axis	Preclinical	[128]
	— *	Structure-based design of the METTL1-WDR4-tRNA complex	Preclinical	[129,130]
eIF4E	Aspirin	Inhibiting PI3K/Akt/mTORC1 signaling and suppressing eIF4E activity	Preclinical	[101]
	ISIS 183750	Antisense oligonucleotide targeting eIF4E	Phase I/II clinical trial	[131]
	4EGI-1	Disrupting eIF4E-eIF4G interaction and inhibiting eIF4F complex assembly	Preclinical	[132]
	Sizofiran	Potential eIF4E inhibitor identified by computational studies	Preclinical (in silico)	[134]
	Ribavirin	Functional mimetic of eIF4E and competitively inhibiting eIF4E activity	Preclinical	[135,136]
	eFT508	MNK inhibitor; reducing p-eIF4E (S209) levels	Phase II clinical trial	[138]
	ETC-206	MNK inhibitor; reducing p-eIF4E (S209) levels	Phase II clinical trial	[138]
	Rapalogs	mTOR inhibitors; reducing p-4E-BP1 levels	Preclinical	[139]
	NVP-BEZ235	Dual PI3K/mTOR inhibitor	Preclinical	[139]
	Metformin	Inhibiting both mTOR and MNK1 signaling and suppressing eIF4E activity	Preclinical	[141]

* No suitable clinical candidate identified.

## Data Availability

No new data were created or analyzed in this study. Data sharing is not applicable to this article.
